# BMP-7 Treatment Increases M2 Macrophage Differentiation and Reduces Inflammation and Plaque Formation in Apo E^-/-^ Mice

**DOI:** 10.1371/journal.pone.0147897

**Published:** 2016-01-29

**Authors:** Dinender K. Singla, Reetu Singla, Jing Wang

**Affiliations:** Division of Metabolic and Cardiovascular Sciences, Biomolecular Science Center, Burnett School of Biomedical Sciences, College of Medicine, University of Central Florida, Orlando, FL, United States of America; Georgia Regents University, UNITED STATES

## Abstract

Inflammation plays a fundamental role in the inception and development of atherosclerosis (ATH). Mechanisms of inflammation include the infiltration of monocytes into the injured area and subsequent differentiation into either pro-inflammatory M1 macrophages or anti-inflammatory M2 macrophages. We have previously published data suggesting bone morphogenetic protein-7 (BMP-7) enhances M2 macrophage differentiation and anti-inflammatory cytokine secretion *in vitro*. In this regard, we hypothesized BMP-7 would inhibit plaque formation in an animal model of ATH through monocytic plasticity mediation. ATH was generated in male and female Apo E^-/-^ mice via partial left carotid artery (PLCA) ligation and mice were divided into 3 groups: Sham, PLCA, and PLCA+BMP-7 (200ug/kg; i.v.). Our data suggest that BMP-7 inhibits plaque formation and increases arterial systolic velocity. Furthermore, we report inhibition of monocyte infiltration and a decrease in associated pro-inflammatory cytokines (MCP-1, TNF-α, and IL-6) in the PLCA+BMP-7 mice. In contrast, our data suggest a significant (p<0.05) increase in M2 macrophage populations with consequential enhanced anti-inflammatory cytokine (IL-1RA, IL-10, and Arginase 1) expression following BMP-7 treatment. We have also observed that mechanisms promoting monocyte into M2 macrophage differentiation by BMP-7 involve the upregulation and activation of the BMP-7 receptor (BMP-7RII). In conclusion, we report that BMP-7 has the potential to mediate cellular plasticity and mitigate the inflammatory immune response, which results in decreased plaque formation and improved blood velocity.

## Introduction

Atherosclerosis (ATH) was once considered a bland lipid storage disease characterized by the deposition of lipids on arterial surfaces leading to restrictive and eventual blockage of normal blood flow with consequential cardiovascular events occurring including myocardial infarction (MI) and stroke. The traditional views of ATH have crumbled with accruing and insurmountable evidence pointing to inflammation as a mediator of all stages of the disease, from its inception and development to the end-stage thrombotic complications [1, 2]. Basic science and growing knowledge, when applied to ATH, affords new insight into the mechanisms underlying inflammatory-driven pathophysiological atherosclerotic events.

In general, normal endothelium does not allow for the adherence of blood leukocytes. However, during inflammation, the endothelial monolayer expresses adhesion molecules, which bind ligands on the leukocytes [3, 4]. Secreted pro-inflammatory cytokines/chemokines, within the atheroma, provide a favorable chemoattractant environment, facilitating the penetration of the adherent leukocytes into the intima. Various cell types are involved in the transmigration including monocytes and lymphocytes, which then perpetuate the inflammatory response. Specifically, following migration, monocytes differentiate into one of two antagonistic macrophage subtypes [5]. M1 macrophages, or “classically activated” macrophages, promote and enhance the inflammatory response while upregulating a host of characteristic pro-inflammatory cytokines including monocyte chemoattractant protein -1(MCP-1), tumor necrosis factor-alpha (TNF-α) and interleukin-6 (IL-6) [5]. Conversely, M2 macrophages, or “alternatively activated” macrophages, are anti-inflammatory and secrete factors such as interleukin-1 receptor antagonist (IL-1RA), interleukin-10 (IL-10) and Arginase 1 [6, 7]. Data have suggested M2 macrophages play an important role in inflammation mediation and resolution with resultant tissue repair and wound healing [8, 9]. In this regard, identification of growth factors and small molecules that mediate monocyte differentiation may offer novel therapeutic options for patients suffering from inflammatory propagated diseases including ATH.

Bone morphogenetic proteins (BMPs), members of the transforming growth factor beta (TGF-β) superfamily, play a role in variegated processes including bone formation, apoptosis, cell fate and differentiation, and embryogenesis [9, 10]. Recently, we published data suggesting BMP-7 has the potential to direct cellular plasticity, specifically monocytes into M2 macrophages, *in vitro* when cultured in “inflammation mimicry” media [9]. However, the role of BMP-7 in inflammation mediation in the context of ATH had never been explored. In this study, ATH was generated in Apo E^-/-^ mice by partial left carotid artery (PLCA) ligation and following BMP-7 treatment, plaque formation, monocyte infiltration, M1/M2 macrophage differentiation outcomes, pro- and anti-inflammatory cytokine expression, BMP-7 receptor (BMP-7RII) expression on monocytes and M2 macrophages, and blood flow was evaluated. To the best of our knowledge, this is the first report to suggest BMP-7 inhibits plaque formation, monocyte infiltration, and pro-inflammatory cytokine secretion as well as enhances M2 macrophage differentiation, anti-inflammatory cytokine secretion, BMP-7R expression on monocytes and M2 macrophages, and systolic velocity in a PLCA mouse model of ATH.

## Materials and Methods

### Partial Left Carotid Artery (PLCA) Ligation

All mice and animal protocols were approved by the University of Central Florida Institutional Animal Care and Use Committee (IACUC) and as previously reported [11]. In brief, male and female Apo E^-/-^ mice between 8 and 10 weeks of age were divided into three groups (n = 8 animals/group): Sham, PLCA, and PLCA+BMP-7 (200ug/kg; i.v.). Mice were sedated with inhalatory 4% isoflurane via nose cone on a temperature controlled heating pad. A 4–5 mm ventral midline incision was made in the neck and the visualized muscles were separated using blunt dissection with curved forceps to expose the left carotid artery (LCA). Using a surgical light microscope, three branches (left external carotid, left internal carotid, and occipital artery) of the LCA were permanently ligated using a 7–0 silk suture. To provide a source of blood circulation, the superior thyroid artery was left intact. The sham group underwent all aforementioned procedures with the exception that they received a loose suture around the LCA instead of a complete ligation. The incision was then sutured and the mouse remained on the heating pad until consciousness was regained. Following surgery, animals were injected intravenously with BMP-7 (200ug/kg of body weight). Injections were repeated on 2 subsequent days (total of 3 injections) and mice were left to recover for 14 days following last injection.

### Preparation of Paraffin Sections and Histopathology

The left and right common carotid artery, the trachea, and the esophagus were embedded in paraffin to prepare a tissue embedded block. Serial sections (5μm) were made using a tissue processor and then placed on microscope slides (Fisher Scientific, USA). Cross sections were stained using the standard protocol for Masson’s trichrome staining to visualize plaque area under a bright field light Olympus microscope. LCA images were taken and plaque area was measured using Image-J software as previously published [11].

### Immunohistochemistry

To determine the presence of infiltrated monocytes and M1 and M2 macrophages in the plaque, carotid artery sections, prepared using our standard protocol, were boiled in retrieval buffer for 20 min following deparaffinization in xylene [11]. Once cooled to room temperature, the sections were washed in PBS and blocked in 10% normal goat serum (NGS). Sections were then incubated overnight at 4°C with primary antibodies against either CD14 (monocytes, Abbiotec, #25156, 1:50), iNOS (M1 macrophages, Abcam, #ab129372, 1:50), or CD206 (M2 macrophages, Abcam, #ab64693, 1:50). To detect BMP-7 receptor (BMP-7R) expression on monocytes and M2 macrophages, previously stained CD14 and CD206 sections were double stained with BMPRII (Santa Cruz, #sc-5683, 1:50). Following washings in PBS, slides were incubated with appropriate secondary antibodies for 1 hr at room temperature. Sections were mounted with Vectashield containing DAPI (4’, 6-diamidino-2-phenylindole) (Vector Labs) and cover-slipped. LCA artery images (1–2 sections, n = 5 animals/group) were taken for data quantification using an Olympic IX-70 fluorescent microscope and representative images were captured using a confocal microscope. The % monocytes/artery was calculated by counting total CD14 positive cells on the artery/total arterial DAPI x 100. The % M1 macrophages/artery was calculated by counting total iNOS positive cells on the artery/total arterial DAPI x 100. The % M2 macrophages/artery was calculated by counting total CD206 positive cells on the artery/total arterial DAPI x 100. The % of BMPRII on monocytes was calculated by counting double positive CD14/BMPRII cells on the artery/total CD14 positive cells x 100. The % of BMPRII on M2 macrophages was calculated by counting double positive CD206/BMPRII cells on the artery/total CD206 positive cells x 100.

### Pro- and Anti-Inflammatory Cytokine Analysis via ELISA

Pro-inflammatory cytokines (RayBiotech, MCP-1 #ELM-MCP-001 and TNF-α #ELM-TNF-α-001), anti-inflammatory cytokines (RayBiotech, IL-10 #ELM-IL10-001 and IL-1RA #ELM-IL1RA-001), and BMP-7 expression (TSZ Scientific, #M7485) were quantified using commercially available ELISA kits, per manufacture’s instructions, on blood serum samples previously collected from each animal. In brief, blood from each animal was collected post-sacrifice in EDTA coated tubes and centrifuged at 13000 rpm for 15 min. Supernatant containing serum was collected into separate tubes and stored at -20°C for future analysis. For each ELISA, serum was loaded into pre-coated 8-well strips for 2.5 hrs at room temperature. After washing, the detection antibody (biotinylated anti-mouse antibodies) was added followed by HRP-conjugated streptavidin and TMB One-Step substrate reagent, as provided in the kit. When the blue color developed, stop solution was added and the wells were measured at 450 nm in a microtiter plate reader (Bio Rad). Results were normalized to protein concentration and data is presented as arbitrary units (A.U.).

### RT-PCR

Total RNA was isolated from carotid artery homogenates using TRI^®^ Reagent RNA Isolation Reagent (Sigma-Aldrich) and retro transcribed into cDNA using iScript^™^ Select cDNA Synthesis Kit (Bio-Rad). Real-time PCR was carried out with a C1000^™^ Thermal Cycler (Bio-Rad) using SYBR Green (Bio-Rad). TNF-α, IL-6, and Ariginase-1 (Arg1) were normalized against GAPDH. Primers used for RT-PCR were as follows:

TNF- α: sense primer, 5′-CACAGAAAGCATGATCCGCGACGT-3′;

Antisense primer, 5′-CGGCAGAGAGGAGGTTGACTTTCT-3′;

IL-6: sense primer, 5′-TCCAGTTGCCTTCTTGGGAC-3′;

Antisense primer, 5′-GTACTCCAGAAGACCAGAGG-3′;

Arg1: sense primer, 5′-CTCCAAGCCAAAGTCCTTAGAG-3′;

Antisense primer, 5′-AGGAGCTGTCATTAGGGACATC-3′;

GAPDH: sense primer, 5′-AACGACCCCTTCATTGAC-3′;

Antisense primer, 5′-TCCACGACATACTCAGCAC-3′;

Final data are presented as relative fold change against sham.

### Systolic Velocity

A Phillips Sonos 5500 Ultrasound system was used to assess systolic blood velocity of the left common carotid artery at D14 post ligation. Briefly, mice were anesthetized under 2–4% isoflurane and 2% oxygen via nose cone. Two-dimensional (2D) images were recorded in the long axis projection using a guided B-mode with the ultrasound probe lying on the left side of the neck. Under Doppler vascular settings, three to four 2D images for systolic (SV; cm/s) blood velocity were obtained and averaged.

### Statistical Analysis

Data was analyzed using One-way analysis of variance (ANOVA) followed by the Tukey Test. Data is presented as a mean ± SEM and statistical significance assigned when p<0.05.

## Results

### BMP-7 Inhibits Atherosclerotic Plaque Formation in Apo E^-/-^ Mice

**Fig 1:**
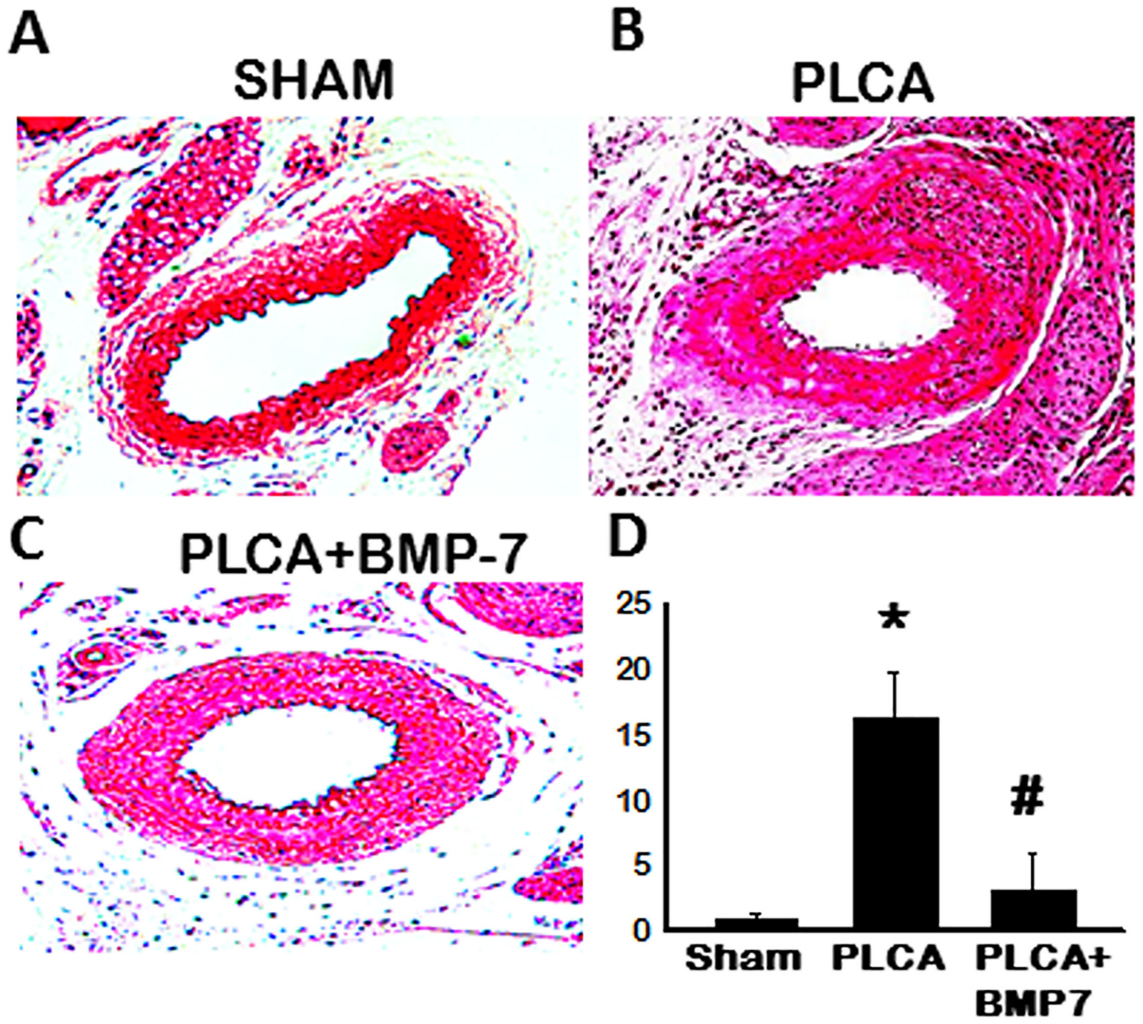
BMP-7 Inhibits Plaque Formation Following PLCA Ligation. **A-C**: Representative photomicrographs of Masson’s trichrome stained arterial sections from all control and experimental groups. **D**: Bar graph of quantitative data suggests plaque formation is inhibited following BMP-7 treatment. *p<0.05 vs. sham and #p<0.05 vs. PLCA.

Masson’s trichrome stained sections were examined to determine the extent of plaque formation as a determinant of atherosclerotic lesions in all control and experimental groups (Fig 1A–1C). Quantitative analysis, at two weeks following permanent PLCA ligation, shows a significant increase in plaque formation in the PLCA group relative to sham controls (p<0.05, Fig 1D). However, when treated with BMP-7, the extent of plaque formation was significantly abrogated compared with the PLCA group (p<0.05, Fig 1D).

### BMP-7 Inhibits Monocyte Infiltration Following PLCA Ligation

**Fig 2:**
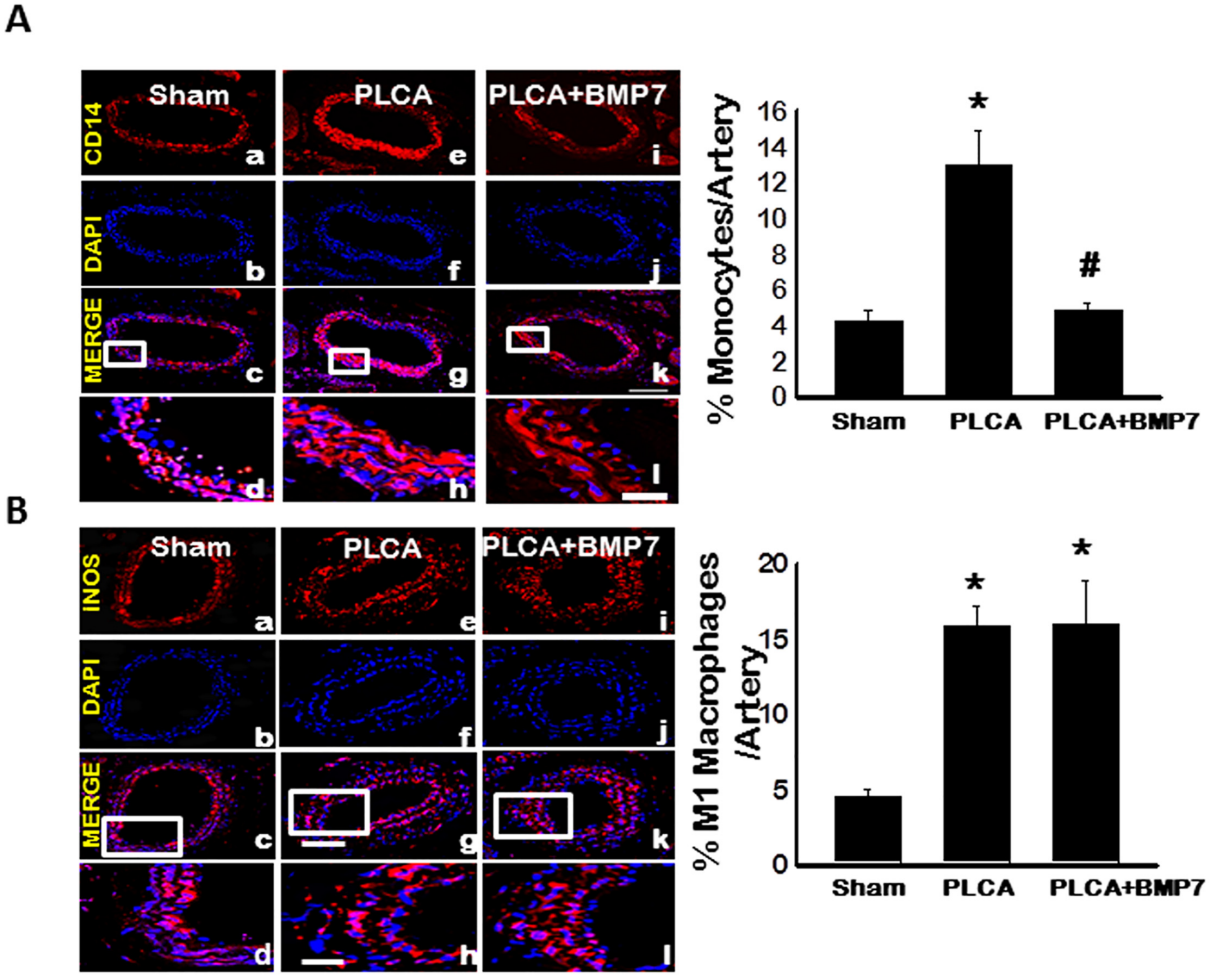
BMP-7 Inhibits Monocyte Infiltration in ATH Apo E^-/-^ Mouse Model. **A**: Representative photomicrographs of carotid artery sections depicting CD14^+ve^ cells, a marker for monocytes, in red (a, e, and i), DAPI, for nuclei, in blue (b, f, and j), merged images (c, g, and k), and enhanced boxed merged images (d, h, and l). Quantitative data suggests monocytic infiltration is significantly diminished following BMP-7 treatment. Scale bars in k and l = 100 μm and 25 μm, respectively. **B**: Representative photomicrographs of carotid artery sections depicting iNOS^+ve^ cells, a marker for M1 macrophages, in red (a, e, and i), DAPI, for nuclei, in blue (b, f, and j), merged images (c, g, and k), and enhanced boxed merged images (d, h, and l). Right bar graph of quantitative analysis suggest M1 macrophage differentiation is unremarkable between PLCA and PLCA+BMP-7 groups. Scale bars in g and h = 100 μm and 25 μm, respectively. *p<0.05 vs. sham and #p<0.05 vs. PLCA.

To extrapolate mechanisms by which BMP-7 inhibits plaque formation post-PLCA ligation, inflammatory cells, specifically monocytes, were quantified. Fig 2A contains representative photomicrographs of carotid artery sections stained with CD14, a marker for monocytes, in red (a, e, and i), DAPI, for nuclei, in blue (b, f, and j), merged images (c, g, and k), and enhanced merged images (d, h, and l). Quantitative analysis suggests a significant upregulation of infiltrated monocytes following PLCA ligation relative to the sham group (p<0.05, Fig 2A). Conversely, monocyte infiltration was significantly reduced upon BMP-7 treatment compared to the PLCA group (p<0.05, Fig 2A).

### M1 Macrophage Differentiation Enhanced Post-PLCA Ligation in Apo E^-/-^ Mice

To evaluate monocytic reprogramming outcomes post-infiltration into the atherosclerotic lesions, we quantified the amount of differentiated pro-inflammatory M1 macrophages. To demonstrate M1 macrophages, representative photomicrographs are shown in Fig 2B depicting iNOS positive M1 macrophages in red (a, e, and i), total nuclei in blue (b, f, and j), merged images (c, g, and k), and enhanced merged images (d, h, and l) for control and experimental groups. The number of iNOS positive M1 macrophages was significantly enhanced post-PLCA ligation compared to sham controls (p<0.05, Fig 2B). Moreover, treatment with BMP-7 following PLCA ligation had no effect on the number of differentiated M1 macrophages relative to the PLCA group (Fig 2B).

### Treatment with BMP-7 Diminishes Pro-Inflammatory Cytokine Expression

**Fig 3:**
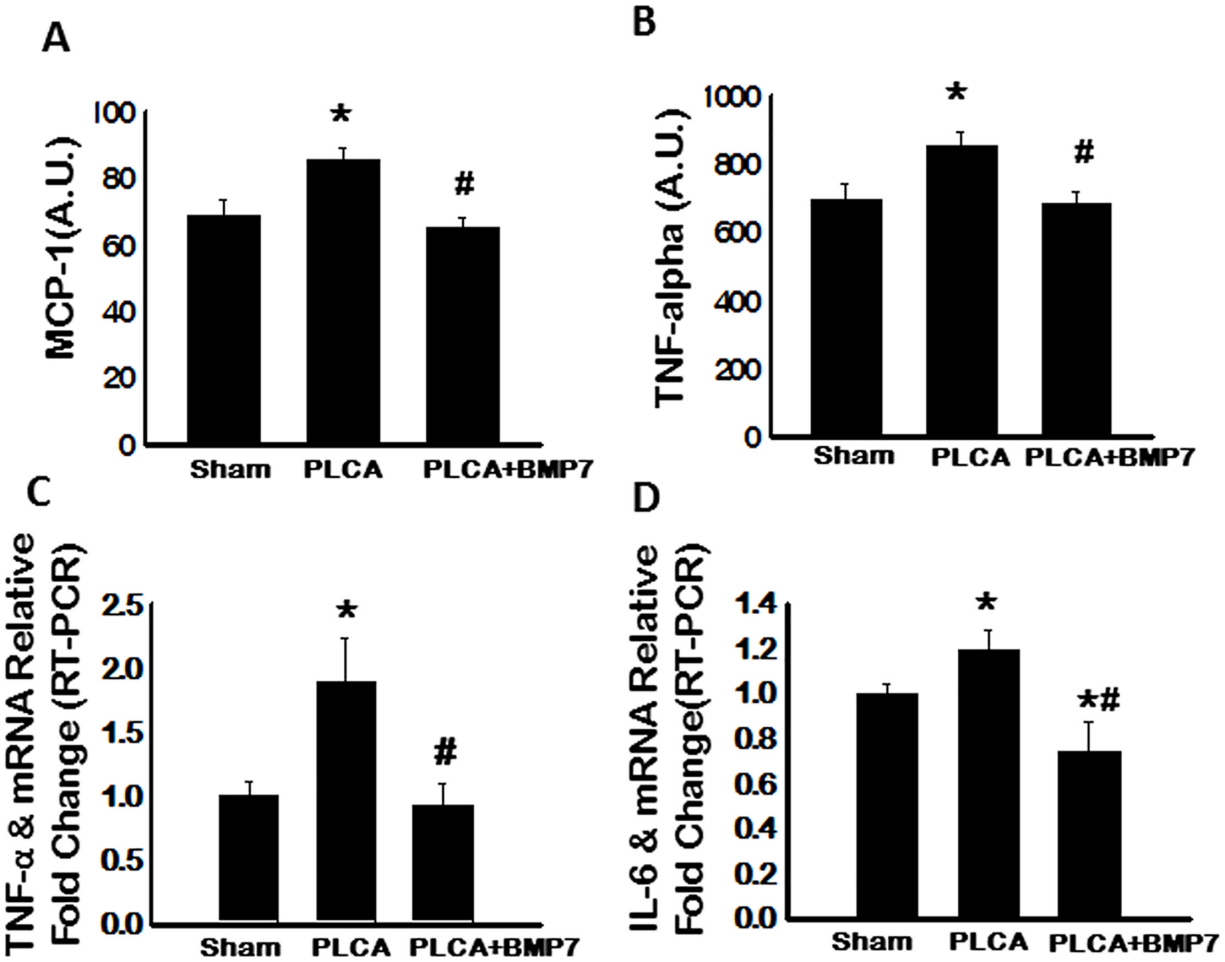
Pro-Inflammatory Cytokine Secretion is Significantly Diminished Following BMP-7 Treatment. **A**: Quantitative analysis of secreted circulatory MCP-1 expression via ELISA **B**: Circulating TNF-α is significantly down regulated post-PLCA ligation with BMP-7 treatment. **C**: Augmented TNF-α expression in PLCA group is confirmed by RT-PCR. **D**: Transcribed IL-6 is significantly enhanced in the PLCA group whereas IL-6 mRNA is significantly diminished following BMP-7 treatment. A. U. = arbitrary units. *p<0.05 vs. sham and #p<0.05 vs. PLCA.

Previous data suggests that macrophage subtypes have unique cytokine profiles with M1 macrophages contributing to enhanced pro-inflammatory MCP-1, TNF-α and IL-6 secretion [5]. In this regard, via ELISA analysis, pro-inflammatory cytokine expression was evaluated and data presented suggest a significant increase in circulating MCP-1 and TNF-α levels following PLCA ligation relative to the sham group (p<0.05, Fig 3A and 3B, respectively). Notably, this increase in MCP-1 and TNF-α expression was significantly abolished following treatment with BMP-7 (p<0.05, Fig 3A and 3B, respectively). Additionally, mRNA levels of TNF-α and IL-6 were evaluated by RT-PCR and our data demonstrates transcribed levels of TNF-αand IL-6 were significantly elevated in the PLCA group relative to sham control animals (p<0.05, Fig 3C and 3D, respectively). Importantly, post-PLCA ligation, administration of BMP-7 significantly blunted both TNF-α and IL-6 mRNA levels compared to the PLCA group (p<0.05, Fig 3C and 3D, respectively).

### M2 Macrophage Differentiation is Enhanced in PLCA Ligated Mice Following BMP-7 Treatment

**Fig 4:**
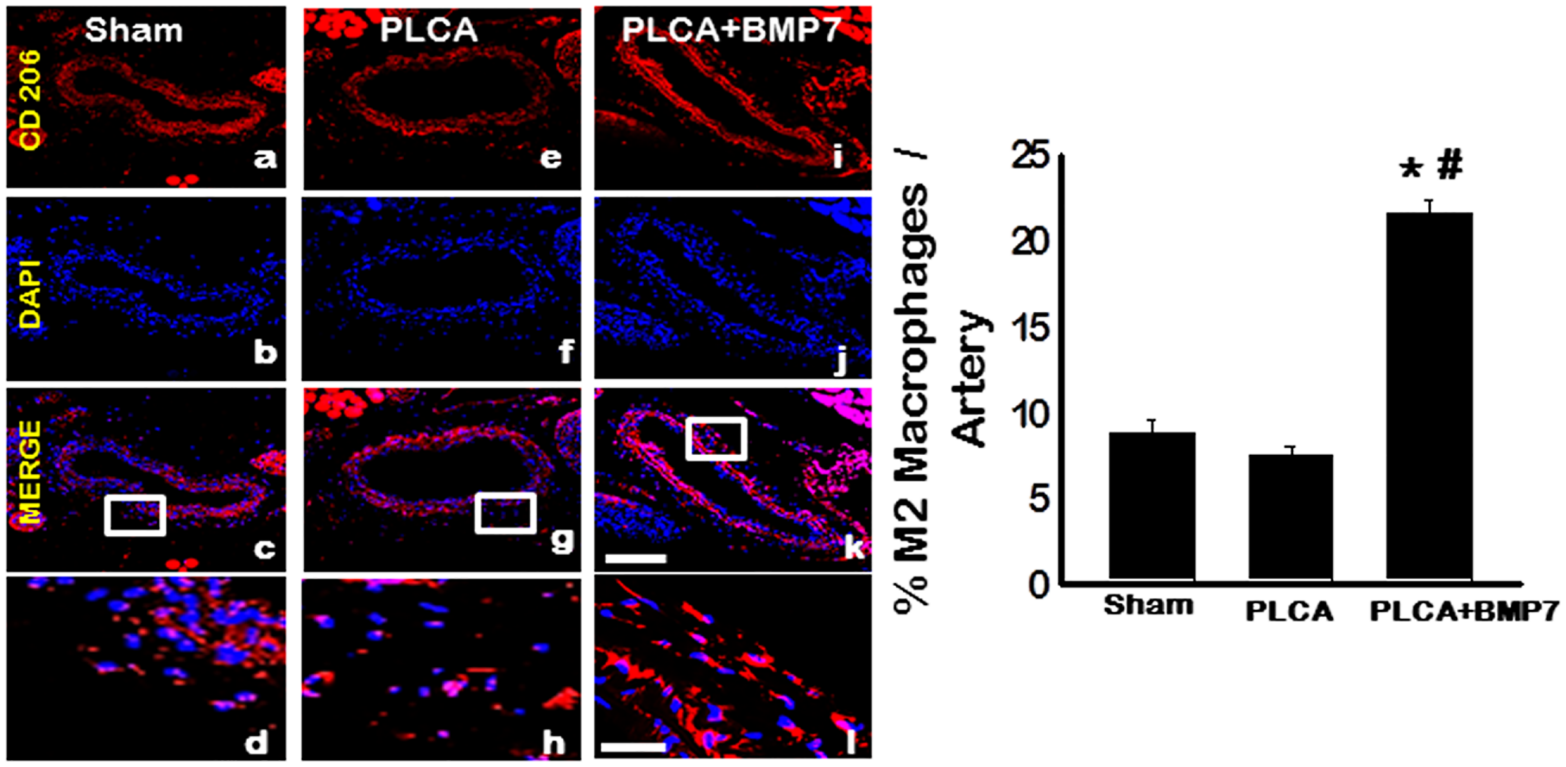
Post-PLCA Ligation, BMP-7 Enhances M2 Macrophage Differentiation. Representative images of LCA sections shown in a-l depict CD206 positive M2 macrophages in red (a, e, and i), total nuclei in blue (b, f, and j), merged images (c, g, and k), and enhanced boxed merged images (d, h, and l). Scale bars in k and l = 100 μm and 25 μm, respectively. Data analysis suggests BMP-7 treatment significantly enhances M2 macrophage differentiation (left bar graph).

Representative images of LCA sections shown in Fig 4a–4l depict CD206 positive M2 macrophages in red (a, e, and i), total nuclei in blue (b, f, and j), merged images (c, g, and k), and enhanced merged images (d, h, and l). Although not statistically significant, a decrease in M2 macrophage was observed in the PLCA group relative to the sham group (Fig 4). Importantly, results showed significantly elevated M2 macrophage concentrations in the PLCA+BMP-7 group relative to sham and PLCA groups, suggesting BMP-7 may play a role in monocyte to M2 macrophage differentiation (p<0.05, Fig 4).

### Anti-Inflammatory Cytokine Expression Increases Following BMP-7 Treatment in PLCA Apo E^-/-^ Mice

**Fig 5:**
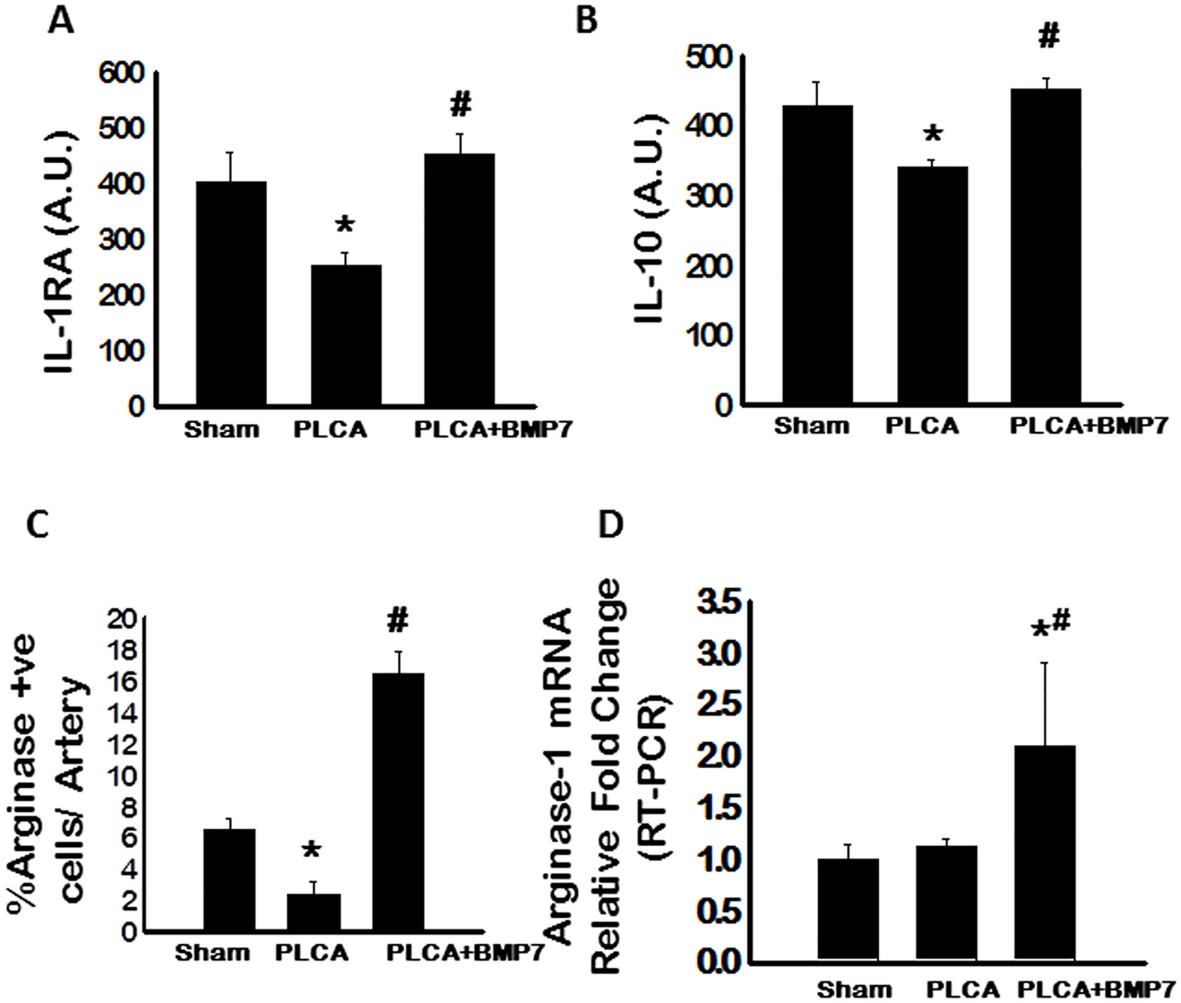
BMP-7 Enhances Circulating Anti-Inflammatory Cytokine Secretion. **A**: Quantitative analysis of secreted circulatory IL-1RA expression via ELISA **B**: Circulating IL-10 is significantly upregulated post-PLCA ligation with BMP-7 treatment. **C**: Bar graph represents immunohistochemistry analysis of Arginase 1 in LCA sections. **D**: Transcribed Arginase 1 is significantly enhanced in the PLCA+BMP-7 group. A. U. = arbitrary units. *p<0.05 vs. sham and #p<0.05 vs. PLCA.

M2 macrophages secrete prototypical anti-inflammatory cytokines including IL-1RA, IL-10, and Arginase 1 [6, 7]. As such, levels of IL-1RA and IL-10 were assessed via ELISA and data presented suggest a significant decline in circulating anti-inflammatory cytokines following PLCA ligation compared to sham (p<0.05. Fig 5A and 5B, respectively). Following treatment with BMP-7, a significant increase in IL-1RA and IL-10 were observed as compared to the PLCA group (p<0.05. Fig 5A and 5B, respectively). Arginase^+ve^ cells within the LCA arteries as well as Arginase 1 mRNA expression were also evaluated. The number of Arginase^+ve^ cells/artery was significantly decreased following PLCA ligation compared to the sham group (p<0.05, Fig 5C). Conversely, the percentage of Arginase^+ve^ cells/artery was significantly enhanced following treatment with BMP-7 (p<0.05, Fig 5C). Moreover, albeit transcribed Arginase 1 mRNA levels were unremarkable between PLCA and sham groups, Arginase 1 mRNA was significantly upregulated in the PLCA+BMP-7 group relative to sham and PLCA groups (p<0.05, Fig 5D).

### Circulating BMP-7 is Diminished Following PLCA Ligation

**Fig 6:**
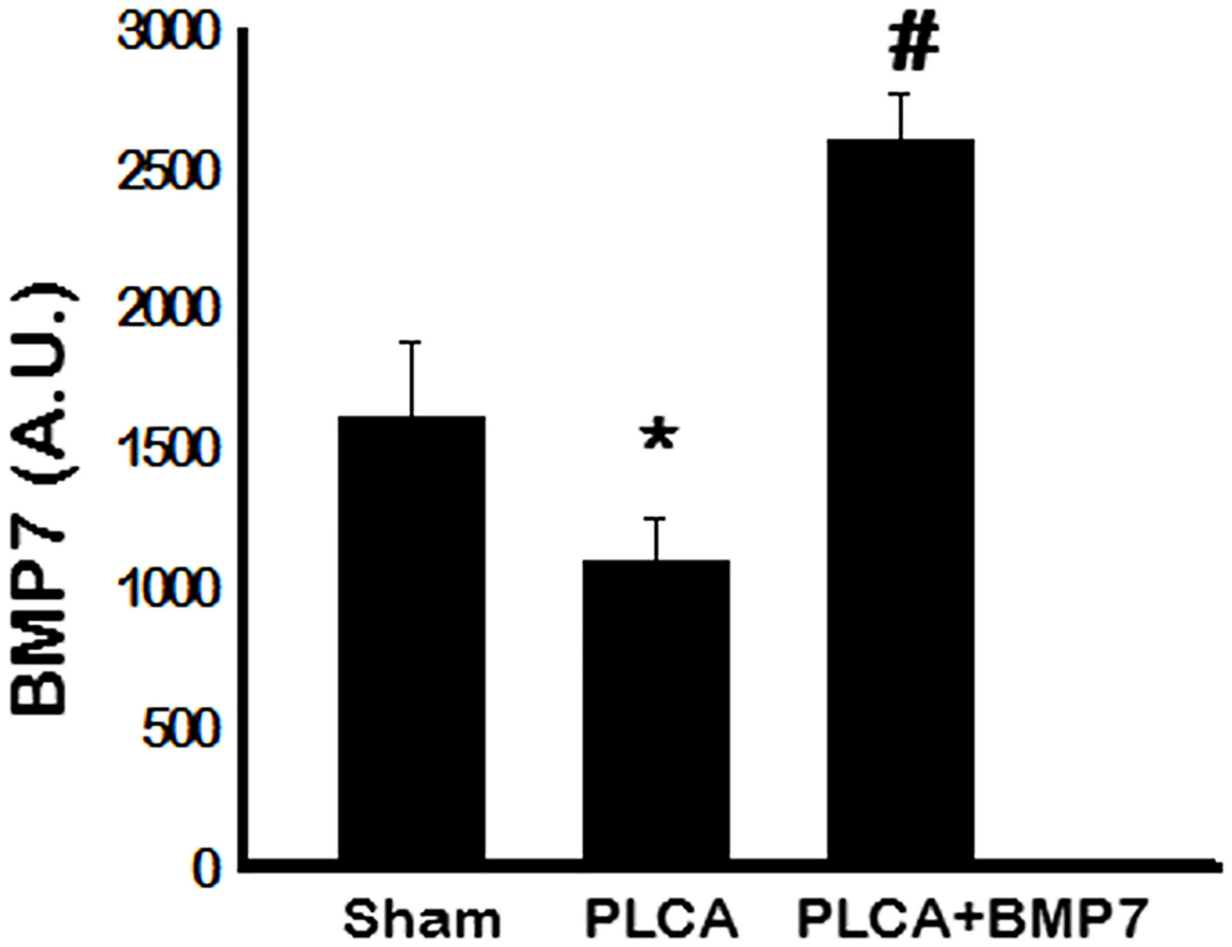
BMP-7 Serum Levels Significantly Diminished Post-PLCA Ligation. Following PLCA ligation, circulating BMP-7 expression was significantly decreased whereas exogenous treatment with BMP-7 significantly enhanced blood serum BMP-7 levels. A. U. = arbitrary units. *p<0.05 vs. sham and #p<0.05 vs. PLCA.

Next, we evaluated the levels of circulating BMP-7 to ascertain a correlation between results obtained and enhanced BMP-7 expression. The BMP-7 expression in the blood was significantly decreased in the PLCA group compared to the sham group suggesting potential interference with normal BMP-7 signaling during ATH (p<0.05, Fig 6). Importantly, following BMP-7 treatment, levels of BMP-7 were significantly upregulated in the PLCA-BMP-7 group relative to the PLCA group (p<0.05, Fig 6).

### BMP-7 Receptor is Upregulated on Monocytes and M2 Macrophages Following BMP-7 Treatment Post-PLCA Ligation

**Fig 7:**
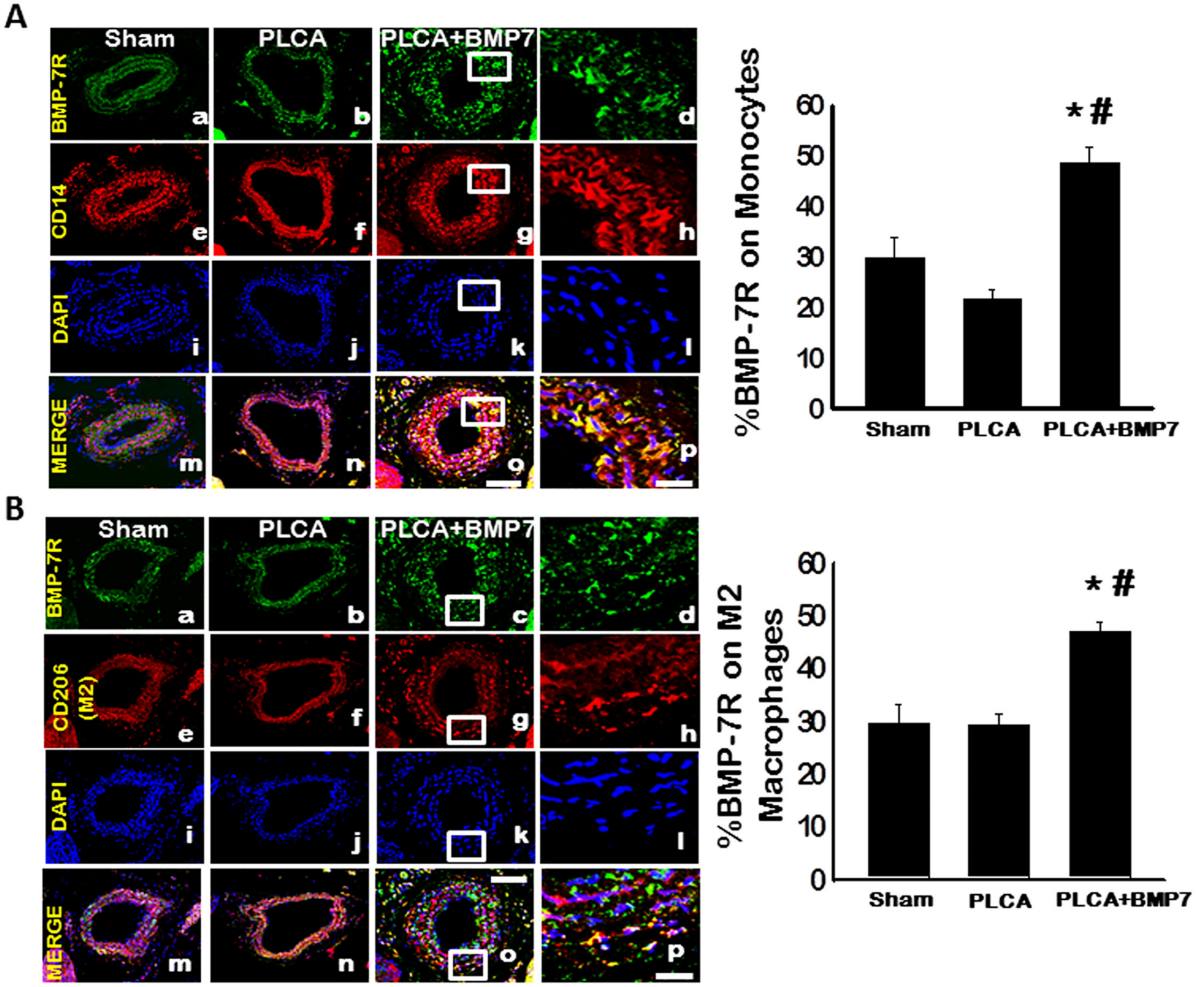
BMP-7 Enhances BMP-7 Receptor Expression on Monocytes and M2 Macrophages. **A**: Representative images of LCAs stained with anti-BMP-7R shown in green (a, b, c, and d), anti-CD14 (monocytes) shown in red (e, f, g, and h), DAPI shown in blue (i, j, k, and l) and merged images (m, n, o and p) are depicted in Fig 7A. Scale bars in o and p = 100 μm and 25 μm, respectively. Data analysis suggests BMP-7 treatment significantly enhances BMP-7R on monocytes (left bar graph). **B**: Representative photomicrographs depict LCAs stained with anti-BMP-7R shown in green (a, b, c, and d), anti-CD206 (M2 macrophages) shown in red (e, f, g, and h), DAPI shown in blue (i, j, k, and l) and merged images (m, n, o and p). Scale bars in o and p = 100 μm and 25 μm, respectively. Data analysis suggests BMP-7 treatment also significantly enhances BMP-7R on M2 macrophages (left bar graph). *p<0.05 vs. sham and #p<0.05 vs. PLCA.

The effects of exogenous BMP-7 on BMP-7 receptor (BMP-7RII) expression on monocytes and M2 macrophages following PLCA ligation were also determined. Representative images of LCAs stained with anti-BMP-7RII shown in green (a, b, c, and d), anti-CD14 (monocytes) or anti-CD206 (M2 macrophages) shown in red (e, f, g, and h), DAPI shown in blue (i, j, k, and l) and merged images (m, n, o and p) are depicted in Fig 7A and 7B. Albeit no difference was observed regarding the percentage of BMP-7R on monocytes between sham and PLCA groups, a significant increase was noted for the PLCA+BMP-7 group relative to both groups (p<0.05, Fig 7A). Additionally, the %BMP-7R on M2 macrophages was also unremarkable between sham and PLCA groups (Fig 7B). However, in the presence of BMP-7, the number of BMP-7 receptors was significantly enhanced on M2 macrophages relative to sham and PLCA ligation operated animals (p<0.05. Fig 7B).

### BMP-7 Enhances Blood Flow Post-PLCA Ligation

**Fig 8:**
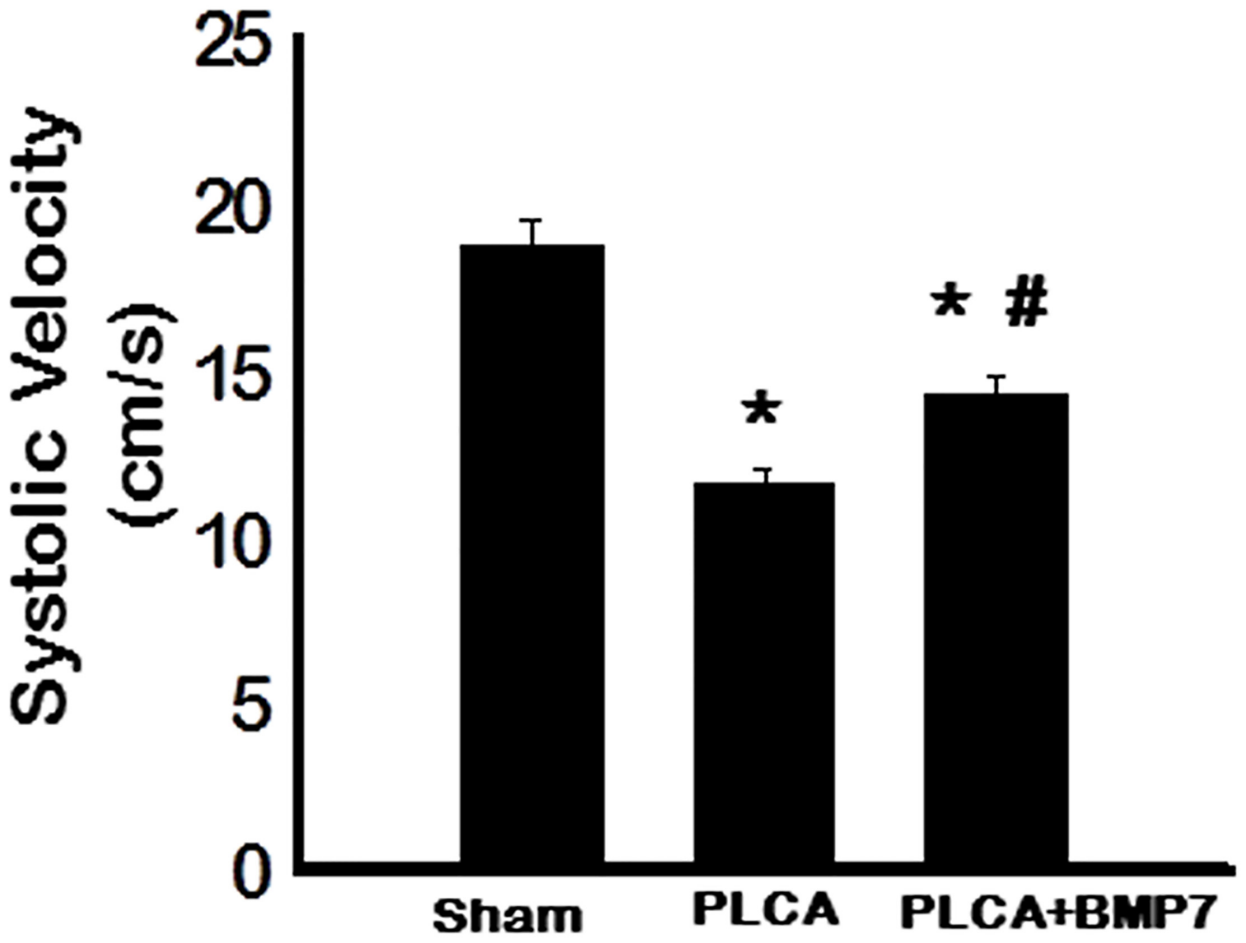
BMP-7 Treatment Enhances Blood Flow Post-PLCA Surgery. Blood flow, measured as systolic velocity, was quantitated two weeks post sham or PLCA ligation. Systolic velocity was significantly decreased post-PLCA ligation but was significantly enhanced when additionally treated with BMP-7. *p<0.05 vs. sham and #p<0.05 vs. PLCA.

Finally, to understand the functional consequences of BMP-7 treatment following PLCA ligation on blood flow, systolic velocity was examined using vascular Doppler ultrasonography. Our data demonstrate a significant reduction in systolic blood flow velocity post-PLCA ligation compared with the sham group suggesting PLCA ligation interfered with normal blood flow and contributed to the development of ATH (p<0.05, Fig 8). Notably, systolic velocity was significantly improved in the PLCA+BMP-7 group compared to the PLCA group but significantly less than that of the sham group (p<0.05, Fig 8).

## Discussion

Atherosclerosis is a progressive disorder characterized by damaged arterial endothelium with consequential plaque formation comprised of low-density lipoproteins (LDLs) and white blood cells (WBCs) that can lead to arterial blockage and normal blood flow interference. Experimental and clinical evidence now provided have highlighted the role of inflammation in the development and progression of atherogenesis [1, 2]. In general, the non-pathogenic arterial endothelium does not support binding of WBCs. However, in early stages of ATH, areas of arterial endothelial cells initiate expression of variegated adhesion molecules including vascular cell adhesion molecule-1 (VCAM-1), which bind various types of leukocytes including monocytes and T lymphocytes. Once adhered to the endothelium, the leukocytes are then able to penetrate the intima mediated by various chemoattractants including MCP-1 [12]. Now infiltrated, the pro-inflammatory WBCs contribute to and promote a local inflammatory response.

Upon infiltration, monocytes undergo reprogramming yielding two antagonistic macrophage phenotypes; “classically activated” pro-inflammatory M1 macrophages and “alternatively activated” anti-inflammatory M2 macrophages. M1 macrophages promote the inflammatory response with concomitant upregulation of various cytotoxic effectors including reactive oxygen/nitrogen intermedites and inflammatory cytokines [6, 7]. Recent reports suggest an association between M1 macrophage differentiation and disease pathophysiology and progression including that of atherosclerosis, cancer, and pre-diabetes [13–16]. Contrarily, M2 macrophages, which encompass all non-classically differentiated macrophages, are implicated in salubrious mediation of the inflammatory response by bolstering remodeling, repair, and resolution, in part, through secretion of quintessential anti-inflammatory cytokines including IL-1RA, IL-10, and Arginase 1 [17, 18]. As such, research is now focused on identification of various growth factors and/or small molecules, which may direct and enhance macrophage plasticity towards the M2 phenotype for the development of novel therapeutic treatments for various diseases. Recently, we reported that BMP-7 enhanced the differentiation of THP-1 monocytes into M2 macrophages with concurrent upregulation of anti-inflammatory cytokines including IL-10 and IL-1RA *in vitro* [9]. However, the role of BMP-7 in inflammatory mediation of atherosclerosis had yet to be investigated. In the present study, we have evaluated the effects of BMP-7 treatment on plaque formation, monocyte infiltration, M1/M2 macrophage differentiation outcomes, pro- and anti-inflammatory cytokine expression, BMP-7R expression on monocytes and M2 macrophages, and blood flow in Apo E^-/-^ mice following PLCA ligation. To the best of our knowledge, this is the first investigation into the salutary inflammatory mediation propagated by BMP-7 in the post-PLCA ligation atherosclerotic model.

Apo E^-/-^ mice, when fed a high fat diet, are regularly used as a model to examine plaque formation and subsequent ATH development, variegated cell infiltration, and vasculature adverse remodeling [19–23]. We, however, have recently published an Apo E^-/-^ mouse ATH model in which ATH was attained by PLCA ligation, in absence of a high fat diet, with evidence of plaque formation, lipid deposition, vascular lesions, thickening of the intima, infiltration of pro-inflammatory cells, and upregulation of pro-inflammatory cytokine release [11]. Using this model, we generated ATH and observed plaque formation was significantly enhanced following PLCA ligation surgery as supported by our previous findings and that of others using high fat diet models of ATH [11, 20, 21, 24, 25]. Importantly, following treatment with BMP-7, plaque formation was significantly reduced. Our data is in accordance with previously published data suggesting that BMP-7 may play a palliative, cytoprotective role in vascular proliferative disorders [26]. Plaque formation and associated infiltrated cell types include monocyte, vascular smooth muscle cells and fibroblasts, etc. The current study addresses the role of monocytes and macrophage differentiation with the use of BMP-7. However, it would be interesting to further examine the effects of BMP-7 on vascular smooth muscle cell proliferation and adventitial fibroblast migration into the arterial intima.

It is widely accepted that ATH is associated with a significant increase in infiltrated monocytes and M1 macrophage polarization, which was also evident in our PLCA ligated mice. However, following BMP-7 treatment, monocytic infiltration was significantly inhibited. BMP-7 administration has been reported to abrogate inflammatory cell infiltrate in other cell types including renal proximal tubular epithelial cells and alkali-injured corneal cells [27, 28]. Although monocyte infiltration was inhibited following BMP-7 administration, it had no effect on M1 macrophage differentiation.

Not only is monocyte/M1 macrophage differentiation implicated in inflammatory disease progression, but also the enhanced expression of various pro-inflammatory chemokines/cytokines [6]. Post PLCA ligation, prototypical M1 macrophage cytokine profiles, including MCP-1, TNF-α, and IL-6, were significantly elevated [5]. However, pro-inflammatory cytokine expression was significantly diminished following treatment with BMP-7 in the post-PLCA ligated mouse. This data is supported by us and others showing BMP-7 inhibits pro-inflammatory cytokine expression *in vitro* and *in vivo* [9, 27, 29–31].

To elucidate mechanisms by which BMP-7 inhibited plaque formation following PLCA ligation surgery, M2 macrophage differentiation and anti-inflammatory cytokine release were evaluated. In the ATH model mice, M2 macrophage populations were unremarkable and anti-inflammatory cytokine expression including IL-1RA, IL-10, and Arginase 1 were significantly decreased compared to sham operated mice. Importantly, following treatment with BMP-7 post-PLCA ligation, M2 macrophage differentiation was significantly enhanced compared to sham and PLCA groups and anti-inflammatory cytokine secretion, including IL-1RA, IL-10, and Arginase 1, was significantly upregulated compared to the PLCA ligated mice. Our data suggest that BMP-7 has the potential to inhibit the pro-inflammatory response that is persistent in ATH through enhanced M2 macrophage differentiation and associated anti-inflammatory mediators as previously evidenced in *in vitro* and *in vivo* models including pre-diabetic cardiomyopathy and inflammatory arthritis [9, 32, 33].

BMP signal transduction, in general, is propagated through ligand induced activation and oligomerization of BMP receptors (BMPRs) which in turn induces phosphorylation and activation of Smad 1/5/8 and Smad-independent cascades regulating target gene transcription [10, 29, 34, 35]. Specifically, the BMPR2 (identified as BMP-7R within the current manuscript) is directly involved in BMP-7 ligand induced receptor activation and oligomerization and subsequent signal transduction activities. We have previously identified a correlation between BMP-7 and upregulated BMP-7R during “inflammation mimicry” assault *in vitro*, which resulted in decreased monocytic populations and enhanced M2 macrophage differentiation [29]. As such, BMP-7R expression was also assessed and its expression remained unchanged on monocytes and M2 macrophages for both sham and PLCA ligated animals as evidenced by co-localization of BMP-7R/CD14 and BMP-7R/CD206, respectively. However, BMP-7 application resulted in a significant increase in BMP-7R expression on both monocytes and macrophages suggesting a correlation between BMP-7R expression and enhanced BMP-7 signal transduction activity. Although we have shown *in vitro* that BMP-7 upregulates BMP-7R expression with consequential phosphorylation and activation of Smad 1/5/8, PI3K, Akt, and mTOR leading to enhanced M2 macrophage differentiation, future studies are warranted to extrapolate the exact signaling cascades mediated by BMP-7, which promote the salutary anti-inflammatory effects in ATH [29].

Finally, blood flow was assessed for all groups and as expected, systolic velocity was significantly disrupted following PLCA ligation. However, following BMP-7 treatment, blood flow was significantly improved in PLCA ligated Apo E^-/-^ mice. Collectively, presented data suggest that BMP-7 inhibits plaque formation and increases arterial systolic velocity in a PLCA ligation model of ATH through inhibition of monocyte infiltration and associated pro-inflammatory cytokines and induction of M2 macrophage differentiation with consequential enhanced anti-inflammatory cytokine expression. Furthermore, we suggest that mechanisms promoting monocyte polarization and M2 macrophage differentiation by BMP-7 involve the upregulation and activation of BMP-7R. Future studies are necessary to understand the involvement of BMP-7R-associated downstream signaling pathways.
